# Effect of a Rubidium Chloride Carrier Confinement Layer on the Characteristics of CsPbBr_3_ Perovskite Light-Emitting Diodes

**DOI:** 10.1186/s11671-021-03641-9

**Published:** 2022-01-03

**Authors:** Chi-Ta Li, Kuan-Lin Lee, Sea-Fue Wang, Lung-Chien Chen

**Affiliations:** 1 Department of Materials and Mineral Resources Engineering, National Taipei University of Technology, 1, Sec. 3, Chung-Hsiao E. Rd., Taipei, 106 Taiwan; 2 Department of Electro-optical Engineering, National Taipei University of Technology, 1, Sec. 3, Chung-Hsiao E. Rd., Taipei, 106 Taiwan

**Keywords:** CsPbBr_3_, RbCl carrier confinement layer, Perovskite, Light-emitting diodes

## Abstract

This work describes the effect of a rubidium chloride (RbCl) interlayer in CsPbBr_3_ perovskite light-emitting diode (LED) structures. RbCl crystallites exhibited polyhedral structures and lattice parameters similar to those of CsPbBr_3_ perovskite crystallites. The lattice mismatch between the RbCl interlayer and CsPbBr_3_ active layer was only approximately 2%. The devices exhibited the best quality and performance when RbCl was used as the nucleation and carrier confinement layer. The crystallite sizes of CsPbBr_3_ with 0.2-, 0.5-, and 1-nm-thick RbCl bottom layers were 55.1, 65.4, and 55.1 nm, respectively. The full width at half maximum (FWHM) of the photoluminescence (PL) emission peak for CsPbBr_3_ with the RbCl bottom layer was 0.096 eV.

## Background

Halide perovskite materials have been extensively studied and are often applied in different photocatalytic and photovoltaic devices owing to their high absorption coefficient and low-cost processing [1–6]. Perovskite is a light-emitting diode (LED) material that shows potential for use as a display light source in future because of its impressive properties, including high color purity, high photoluminescence quantum yield, and low nonradiative recombination ratio [7–9]. A common engineering method used to boost the luminance of perovskite LEDs is to modify the interfaces between the cathode, electron transport layer, perovskite active layer, hole transport layer, and anode, thereby maximizes carrier injection to the fullest extent possible, except for optimizing the perovskite grain size and conductivity of the layers.

Rubidium chloride (RbCl) has been used as an electron transport layer (ETL) with organic light-emitting diodes (OLEDs) to increase electroluminescent efficiency by lowering the effective electron-injecting barrier height and enhancing the electron–hole pair recombination rate [10–12]. Alkali metal cations have also been used to prevent phase segregation of perovskite materials and dopants in PEDOT:PSS used in perovskite solar cell structures, thereby modifying the interface between PEDOT:PSS and the perovskite active layer [13–15]. In addition, RbCl has been employed with the solution method to dope CsPbBr_3_ perovskite films used for blue LEDs [16]. However, interface engineering is important for optoelectronic device structures. Therefore, in this work, we investigate the effect of the RbCl layer used as an ETL in CsPbBr_3_ perovskite light-emitting diodes to improve carrier injection and the buffer layer between PEDOT:PSS and CsPbBr_3_ perovskite active layers to increase the power conversion efficiency.

## Methods

### Materials and Syntheses

CsPbBr_3_ perovskite LEDs were fabricated on indium tin oxide (ITO)-coated glass substrates (Ruilong Ltd., Taiwan). Cesium bromide (CsBr, 99.99%), lead iodide (PbI_2_, 98%), dimethyl sulfoxide (DMSO, 99.9%), rubidium chloride (RbCl, 99.8%), polyethylene oxide (PEO, Mv 400,000), and 1,3,5-benzinetriyl)-tris(1-phenyl-1-H-benzimidazole (TPBi, 99%) were obtained from UniRegion Bio-Tech Ltd. (Taiwan).

First, a CsPbBr_3_ perovskite solution was prepared: 96 mg of cesium bromide (CsBr) and 110 mg of lead bromide (PbBr_2_) were dissolved in 1 mL of dimethyl sulfoxide (DMSO) and then stirred and degassed at 70 °C overnight to produce solution A. Then, 10 mg of polyethylene oxide (PEO) was dissolved in 1 mL of DMSO and stirred and degassed at 70 °C overnight to produce solution B. Then, 0.5 mL of solution A and 20 mg of TPBi powder were added to solution B and stirred until it became completely transparent.

### Device Preparation

**Fig. 1:**
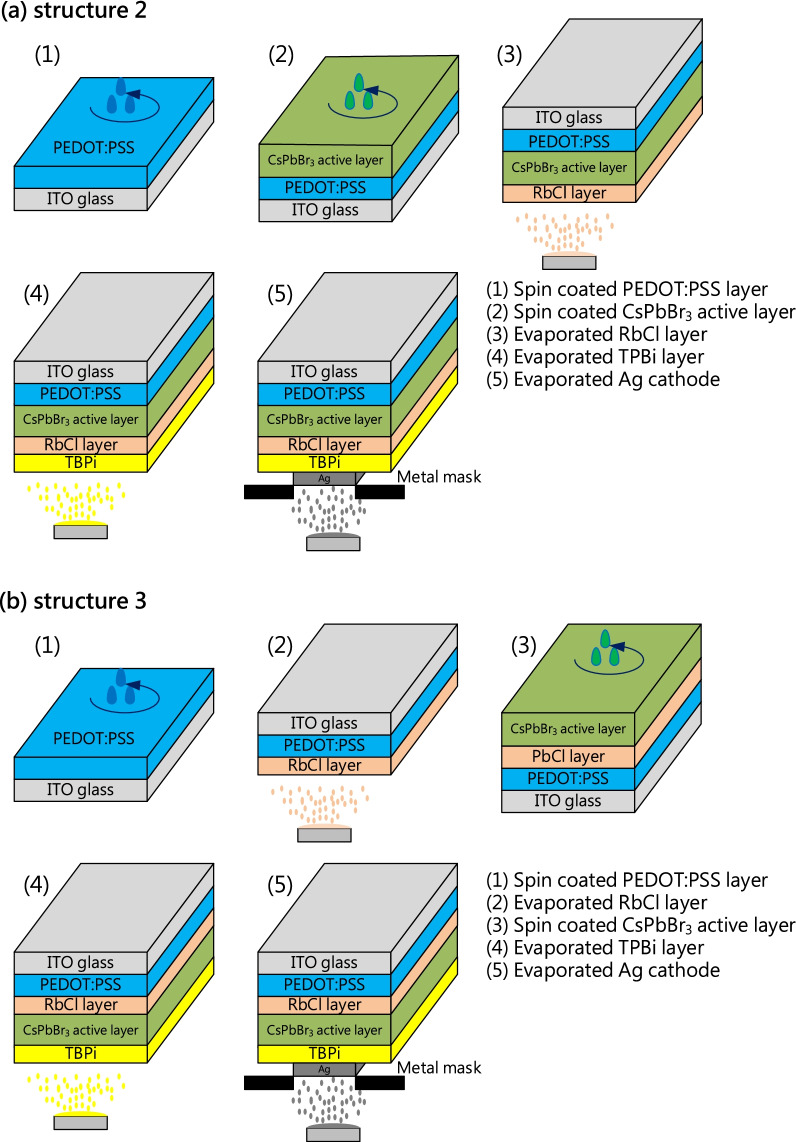
Process flow of CsPbBr_3_ perovskite LEDs with **a** a RbCl upper layer and **b** a RbCl bottom layer

ITO glass substrates were cleaned with acetone, alcohol, and isopropanol by ultrasonic cleaning for 15 min. PEDOT:PSS was spin-coated on the ITO substrate at 5000 rpm for 60 s and then baked at 140 °C for 15 min. For structure 2, the CsPbBr_3_ perovskite solution was spin-coated on the PEDOT:PSS layer at 3000 rpm for 60 s and baked at 80 °C for 10 min. The RbCl upper layer was deposited on the CsPbBr_3_ perovskite layer by the thermal evaporation method. Subsequently, 13-nm-thick TPBi and 100-nm-thick Ag were sequentially deposited by thermal evaporation on the RbCl upper layer. The whole LED structure and process flow are shown in Fig. 1a. For structure 3, the RbCl layer was deposited on the PEDOT:PSS layer by the thermal evaporation method. The CsPbBr_3_ perovskite solution was spin-coated on the RbCl bottom layer at 3000 rpm for 60 s and baked at 80 °C for 10 min. Subsequently, 13-nm-thick TPBi and 100-nm-thick Ag were sequentially deposited on the CsPbBr_3_ perovskite by thermal evaporation. The whole LED structure and process flow are shown in Fig. 1b. In this work, three thicknesses (0.2, 0.5, and 1 nm) were employed for the RbCl layer.

### Characteristics

The morphologies of CsPbBr_3_ films with and without the RbCl layer were observed using field-emission scanning electron microscopy (FESEM, ZEISS Sigma, ZEISS, Munich, Germany). Crystallite sizes and lattice parameters were estimated using X-ray diffraction (X’Pert PRO MRD, PANalytical, Almelo, Netherlands). Optical characteristics were measured by a fluorescence spectrophotometer (F-7000, Hitachi, Tokyo, Japan). The optoelectronic properties of LEDs were measured using a Keithley 2420 source meter and PR-670 spectroradiometer (JADAK, New York, USA). All characterizations were measured at room temperature.

## Results and Discussion

**Fig. 2:**
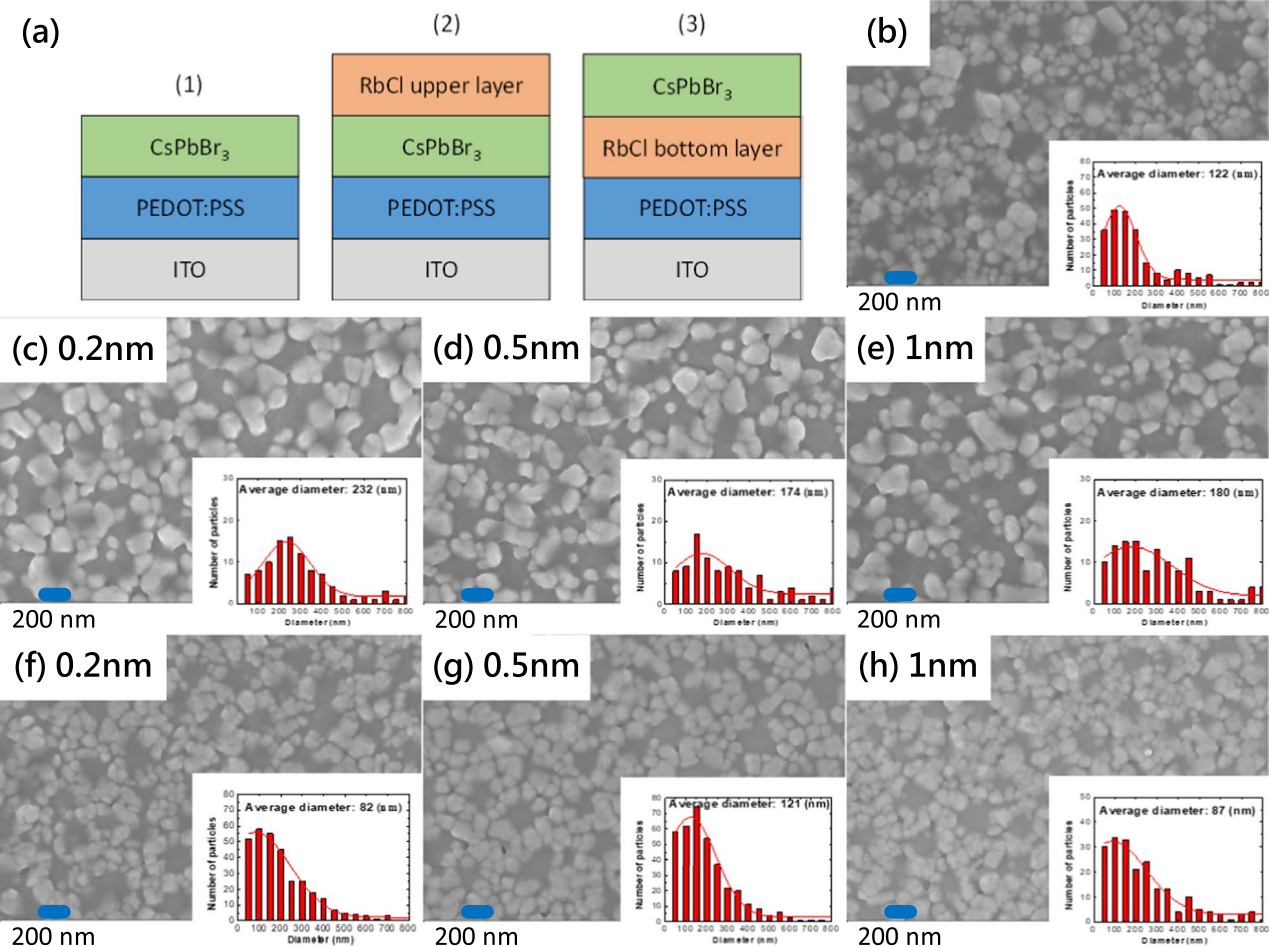
**a** Sketch of the three structures studied in this work. Top-view FESEM images of **b** structure 1, **c**–**e** structure 2 with various RbCl layer thicknesses, and **f**–**h** structure 3 with various RbCl layer thicknesses. The insets in FESEM images are particle size distribution histograms

Three structures were studied in this work, as shown in Fig. 2a. Figure 2b–h show top-view FESEM images of the three structures. Figure 2b shows the morphology of structure 1, which is a control sample without the RbCl layer, for comparison with other structures. Figure 2c–e are top views of structure 2 with various thicknesses of the RbCl upper layer. The particle sizes on the surface are larger than those of the control sample without the RbCl layer. Figure 2f–h are top views of structure 3 with various thicknesses of the RbCl bottom layer. The insets of FESEM images in Fig. 2b–h are particle size distribution histograms. The average particle sizes were 120, 180–230, and 80–120 nm for structures 1, 2, and 3, respectively. The particle on the surface were smaller and denser than those of the control sample without the RbCl layer. This may be because the RbCl bottom layer assists nucleation of the CsPbBr_3_ perovskite film.

**Fig. 3:**
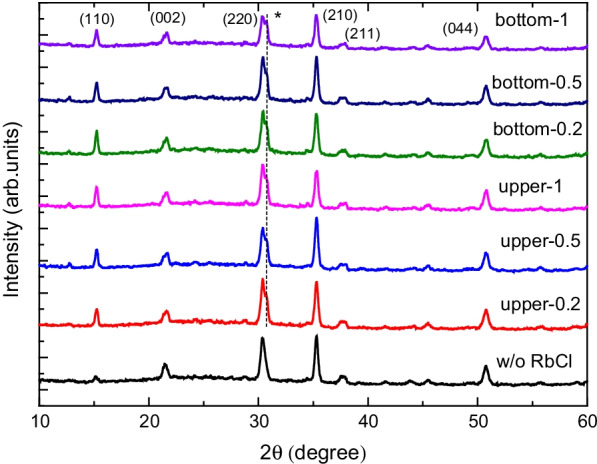
XRD patterns of various structures with different thicknesses of the RbCl layer

Figure 3 presents the XRD patterns for various structures and thicknesses of the RbCl layers. For the structure without a RbCl layer, six clear diffraction peaks at 15.28°, 21.43°, 30.42°, 35.28°, 37.63°, and 50.73° were observed. They correspond to the (110), (112), (220), (210), (211), and (044) planes of orthorhombic CsPbBr_3_ crystallites, respectively [17–19]. For the structure with a RbCl layer, one diffraction shoulder at 30.72° was observed that corresponds to the RbCl dopant due to diffusion effect [20, 21]. The ionic Rb^+^ and Cs^+^ have similar ionic radii and the Rb^+^ substitution could stabilize the orthorhombic CsPbBr_3_ phase, as a resulting of diffraction peak of orthorhombic RbPbBr_3_ [20, 22]. To compare CsPbBr_3_ film quality, crystallite sizes were estimated by Scherrer's equation [23, 24]. The crystallite sizes of CsPbBr_3_ without and with 0.2-, 0.5-, and 1-nm-thick RbCl bottom layers were 53.5, 55.1, 65.4, and 55.1 nm, respectively. Therefore, the RbCl bottom layer improved the CsPbBr_3_ crystallites due to their similar polyhedral structures.

**Fig. 4:**
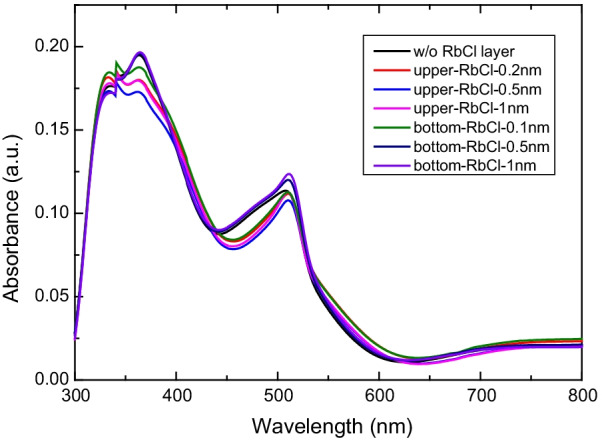
Absorption spectra of CsPbBr_3_ films with and without the RbCl layer

Figure 4 shows absorbance spectra for CsPbBr_3_ films with and without RbCl layers. The absorption edges of all samples appeared at 520 nm in the absorbance spectra. The optical band gap of the samples was calculated to be approximately 2.385 eV. This result is consistent with the band gap of the CsPbBr_3_ film. The band gap of the RbCl layer is approximately 4.8 eV. Thus, it was transparent to visible light. Additionally, a noticeable absorption band was seen at 425 nm and is attributed to absorption by the trans-isomers of 4-(nitrophenyl)azo groups in the PEDOT:PSS layer [25, 26].

**Fig. 5:**
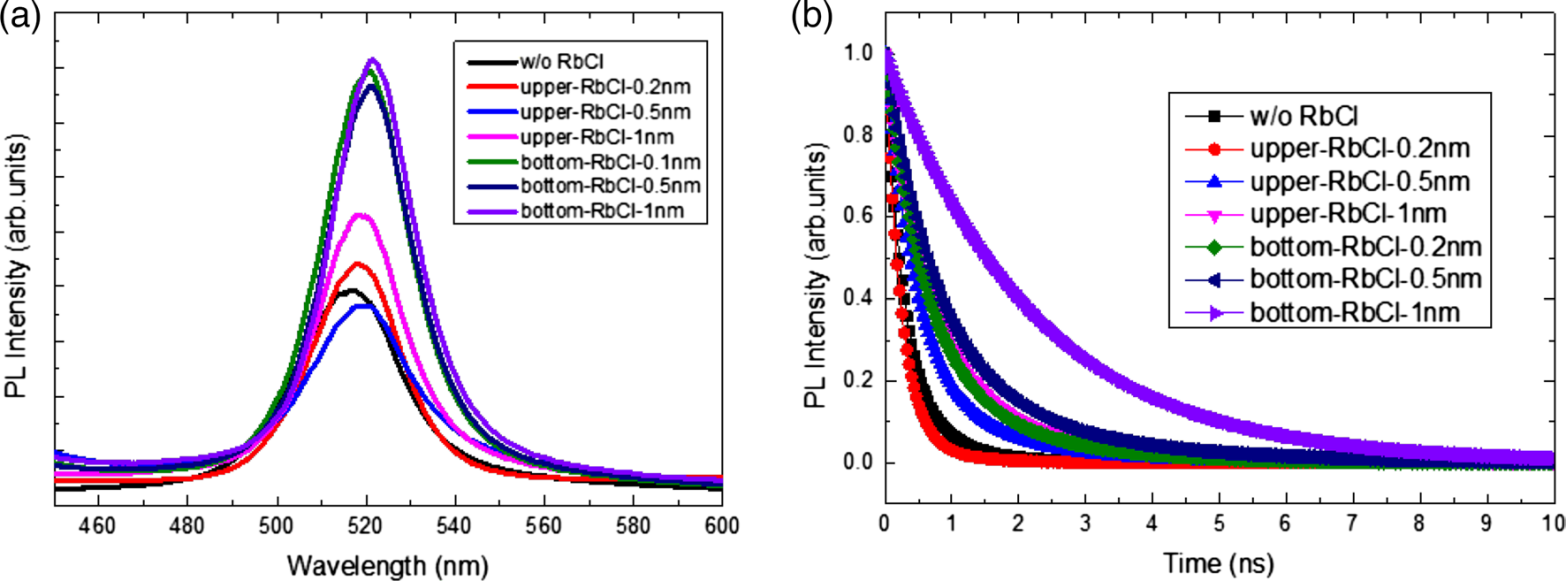
**a** Photoluminescence spectra (PL) and **b** time-resolved PL spectra of the CsPbBr_3_ films with and without a RbCl layer

Photoluminescence (PL) emission spectra of the CsPbBr_3_ films with and without the RbCl layer are shown in Fig. 5a. The emission peaks in the PL spectra of all samples are at 518 nm. This peak corresponds to a band gap of 2.394 eV [27, 28]. Clearly, the intensities for CsPbBr_3_ films with the RbCl bottom layer are higher than those for CsPbBr_3_ film alone and CsPbBr_3_ films with the RbCl upper layer. The PL intensity responds to the carrier lifetime caused by defect-induced recombination. The FWHM of the emission peak of CsPbBr_3_ with the RbCl bottom layer is 0.096 eV. It is less than 0.1 eV for CsPbBr_3_ with the RbCl upper layer and 0.126 eV for CsPbBr_3_ without the RbCl layer owing to the better crystallite quality. The charge carrier recombination kinetics of CsPbBr_3_ films with and without a RbCl layer were investigated by time-resolved photoluminescence (TRPL) measurements, as shown in Fig. 5b. Table 1 lists the parameters obtained from fits of the TRPL spectra. The RbCl bottom layer obviously improved the carrier lifetime, and the carrier lifetime was increased slightly by the RbCl upper layer. In other words, RbCl positioned as the bottom layer of the CsPbBr_3_ film had a better impact than the upper layer on the quality of CsPbBr_3_ films.

**Table 1:** Summary of parameters derived from fitting TRPL spectra

	*A*_1_ (%)	*τ*_1_ (ns)	*A*_2_ (%)	*τ*_2_ (ns)	*τ*_ave_
w/o RbCl	80.83	0.26	19.17	0.75	0.4
upper-RbCl-0.2 nm	92.88	0.23	7.12	0.73	0.3
upper-RbCl-0.5 nm	76.18	0.44	23.82	1.31	0.6
upper-RbCl-1 nm	82.97	0.65	17.03	2.2	0.9
bottom-RbCl-0.2 nm	75.76	0.58	24.24	1.58	0.8
bottom-RbCl-0.5 nm	78.73	0.76	21.27	2.28	1.1
bottom-RbCl-1 nm	50	2.21	50	2.21	2.2

**Fig. 6:**
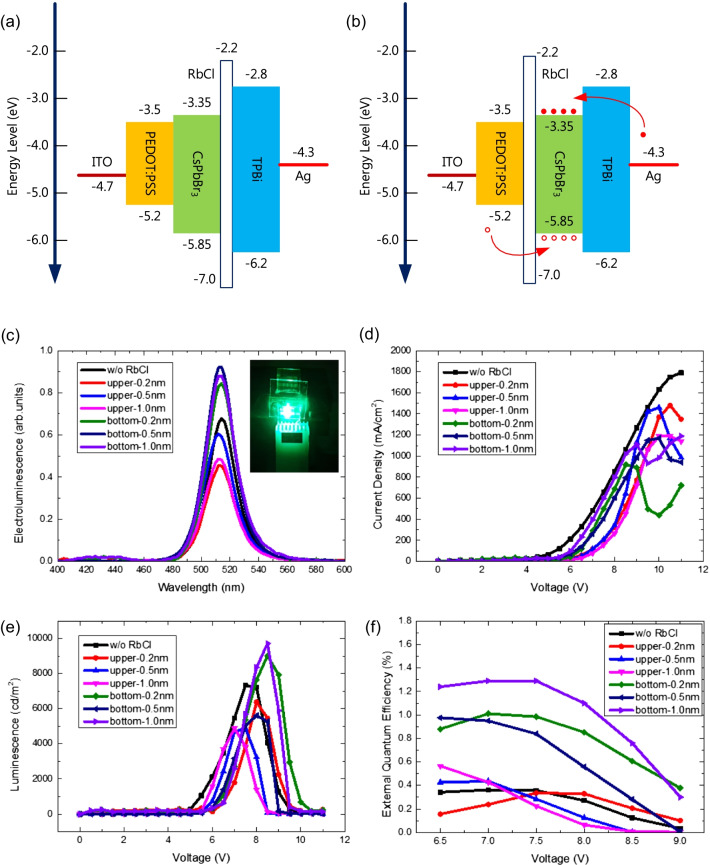
Diagram of the energy levels for **a** structure 2 and **b** structure 3. **c** Electroluminescence (EL), **d** current density, **e** luminescence, and **f** external quantum efficiency of CsPbBr_3_ perovskite LEDs with and without RbCl layers. The inset in (**c**) shows a photo of an operating LED

Figure 6a and b are plots of the energy levels of structure 2 and structure 3. In structure 2, the RbCl layer was formed as an electron transport layer on the CsPbBr_3_ active layer. In structure 3, the RbCl layer was inserted as a carrier confinement layer under the CsPbBr_3_ active layer to improve the efficiency of electron–hole recombination and enhance the performance of CsPbBr_3_ perovskite LEDs [29]. Figure 6c–f plot the electroluminescence (EL), current density, luminescence, and external quantum efficiency of CsPbBr_3_ perovskite LEDs with and without a RbCl layer. Remarkably, the performance of CsPbBr_3_ LEDs with RbCl bottom layers was superior to that of CsPbBr_3_ LEDs without and with a RbCl upper layer owing to improvement in crystallite quality for the CsPbBr_3_ active layer and carrier confinement effect of the RbCl bottom layer, although the turn-on voltage increased from 5 to 5.5 V due to insertion of the RbCl layer, as shown in Fig. 6d. Another factor of performance improvement may be contributed to that Rb^+^ dopant reduces the surface defects and non-radiative recombination of CsPbBr_3_ active layer due to passivation, and then increases the carrier lifetime, as shown in Fig. 5b [16, 20, 21]. As shown in Fig. 6c, the emission peaks for all devices appeared at approximately 514 nm and were consistent with the peaks in the PL spectra, as shown in Fig. 5. The inset of Fig. 6c shows a photograph of an operating LED. The intensities of the EL spectra for CsPbBr_3_ LEDs with RbCl bottom layers were higher than those of CsPbBr_3_ LEDs without and with a RbCl upper layer. The best luminescence and external quantum efficiency were 9718 cd/m^2^ at a bias of 8.5 V and 1.29% at a bias of 7.5 V, respectively. These values constituted improvements of 33% and 262%, respectively. In addition, in this work, CsPbBr_3_ LEDs with RbCl upper layers did not show the effects of an electron transport layer, similar to an OLED structure, according to Fig. 6c–f. It means that the RbCl impossibly behaves as an electron transport layer in perovskite LEDs.

## Conclusions

In summary, the effect of rubidium chloride (RbCl) interlayers in CsPbBr_3_ perovskite structures of light-emitting diodes (LED) was studied. When using RbCl as the nucleation layer, the CsPbBr_3_ perovskite exhibited the best quality and performance. The crystallite sizes of CsPbBr_3_ with 0.2-, 0.5-, and 1-nm-thick RbCl bottom layers were 55.1, 65.4, and 55.1 nm, respectively. The performance of CsPbBr_3_ LEDs with RbCl bottom layers was superior to those of CsPbBr_3_ LEDs without and with RbCl upper layers. The best luminescence and external quantum efficiencies were 9718 cd/m^2^ at a bias of 8.5 V and 1.29% at a bias of 7.5 V, respectively. These values constituted improvements of 33% and 262%, respectively. This may be due to several factors: the crystallite quality of the improved CsPbBr_3_ active layer, stabile CsPbBr_3_ crystalline, surface passivation strategy of the CsPbBr_3_ active layer, and the carrier confinement effect of the RbCl layer.

## Data Availability

All the data are fully available without restrictions.

## Abbreviations

RbCl: Rubidium chloride
CsPbBr_3_: Cesium lead tri-bromide
CsBr: Cesium bromide
PbBr_2_: Lead bromide
DMSO: Dimethyl sulfoxide
PEO: Polyethylene oxide
FESEM: Field-emission scanning electron microscopy
ITO: Indium tin oxide
PEDOT:PSS: Poly (3,4-ethylenedioxythiophene) polystyrene sulfonate
LED: Light-emitting diode
OLED: Organic light-emitting diode

## References

[1] Schanze KS, Kamat PV, Yang P, Bisquert J (2020). Progress in perovskite photocatalysis. ACS Energy Lett.

[2] Teh YW, Chee MK, Kong XY, Yong S-T, Chai S-P (2020). An insight into perovskite-based photocatalysts for artificial photosynthesis. Sustain Energy Fuels.

[3] Kojima A, Teshima K, Shirai Y, Miyasaka T (2009). Organometal halide perovskites as visible-light sensitizers for photovoltaic cells. J Am Chem Soc.

[4] National Renewable Energy Laboratory (2021) Best research-cell efficiencies. Available online: https://www.nrel.gov/pv/assets/pdfs/best-research-cell-efficiencies.20200104.pdf. Accessed 4 Jan 2021

[5] Liu Z, Krückemeier L, Krogmeier B, Klingebiel B, Márquez J, Levcenko S, Öz S, Mathur S, Rau U, Unold T, Kirchartz T (2019). Open-circuit voltages exceeding 1.26 V in planar methylammonium lead iodide perovskite solar cells. ACS Energy Lett.

[6] Gao B, Meng J, Lu J, Zhao R (2020). CH_3_NH_3_PbI_3_ perovskite solar cells with efficiency over 22% fabricated by green antisolvent method. Mater Lett.

[7] Zhang Q, Tavakoli MM, Gu L, Zhang D, Tang L, Gao Y, Guo J, Lin Y, Leung S-F, Poddar S (2019). Efficient metal halide perovskite light-emitting diodes with significantly improved light extraction on nanophotonic substrates. Nat Photon.

[8] Park J, Jang HM, Kim S, Jo SH, Lee TW (2020). Electroluminescence of perovskite nanocrystals with ligand engineering. Trends Chem.

[9] He Q, Mei E, Liang X, Xiang W (2021). Ultrastable PVB films-protected CsPbBr 3/Cs4PbBr6 perovskites with high color purity for nearing Rec. 2020 standard. Chem Eng J.

[10] Lü Z, Deng Z, Du H, Li D, Zou Y, Xu D, Chen Z, Wang Y (2009). The effect of rubidium chloride on properties of organic light-emitting diodes. Solid State Electron.

[11] Lü Z, Wang Y, Zou Y, Du H, Chen Z, Deng Z (2010). The effect of alkaline metal chlorides on the properties of organic light-emitting diodes. J Lumin.

[12] Lü Z, Deng Z, Hou Y, Xu H (2012). Similarities and differences of alkali metal chlorides applied in organic light-emitting diodes. Thin Solid Films.

[13] Zhang S, Tang M-C, Fan Y, Li R, Nguyen NV, Zhao K, Anthopoulos TD, Hacker CA (2020). Role of alkali-metal cations in electronic structure and halide segregation of hybrid perovskites. ACS Appl Mater Interfaces.

[14] Liu X, Li B, Zhang N, Yu Z, Sun K, Tang B, Shi D, Yao H, Ouyang J, Gong H (2018). Multifunctional RbCl dopants for efficient inverted planar perovskite solar cell with ultra-high fill factor, negligible hysteresis and improved stability. Nano Energy.

[15] Wang H, Xu Y, Wu J, Chen L, Yang Q, Zhang B, Xie Z (2020). Bright and color-stable blue-light-emitting diodes based on three-dimensional perovskite polycrystalline films via morphology and interface engineering. J Phys Chem Lett.

[16] Wang HL, Zhao XF, Zhang BH, Xie ZY (2019). Blue perovskite light-emitting diodes based on RbX-doped polycrystalline CsPbBr_3_ perovskite films. J Mater Chem C.

[17] Zhang L, Yuan F, Dong H, Jiao B, Zhang W, Hou X, Wang S, Gong Q, Wu Z (2018). One-step co-evaporation of all-inorganic perovskite thin films with room-temperature ultralow amplified spontaneous emission threshold and air stability. ACS Appl Mater Interfaces.

[18] Tenailleau C, Aharon S, Cohen B-E, Etgar L (2019). Cell refinement of CsPbBr_3_ perovskite nanoparticles and thin films. Nanoscale Adv.

[19] Zhang M, Zheng Z, Fu Q, Chen Z, He J, Zhang S, Yan L, Hu Y, Luo W (2017). Growth and characterization of all-inorganic lead halide perovskite semiconductor CsPbBr_3_ single crystals. Cryst Eng Commun.

[20] Todorović P, Ma DX, Chen B, Quintero-Bermudez R, Saidaminov MI, Dong YT, Lu ZH, Sargent EH (2019). Spectrally tunable and stable electroluminescence enabled by rubidium doping of CsPbBr_3_ nanocrystals. Adv Opt Mater.

[21] Geng YX, Yang BB, Xiang YR, Shi MM, Hu RR, Guo CF, Li YF, Zou J (2021). Preparation and research of perovakite quantum dots power based on RbCl doped CsPbBr_3_. Chem Sel.

[22] Xiao JW, Liang Y, Zhang SY, Zhao YZ, Li YJ, Chen Q (2019). Stabilizing RbPbBr_3_ perovskite nanocrystals through C^s^+ substitution. Chem Eur J.

[23] West AR (2014). Solid state chemistry and its applications.

[24] Chen L-C, Tseng Z-L, Chen C-C, Chang SH, Ho C-H (2016). Fabrication and characteristics of CH_3_NH_3_PbI_3_ perovskite solar cells with molybdenum-selenide hole-transport layer. Appl Phys Exp.

[25] Kalachyova Y, Guselnikova O, Postnikov P, Fitl P, Lapcak L, Svorcik V, Lyutakov O (2018). Reversible switching of PEDOT:PSS conductivity in the dielectric–conductive range through the redistribution of light-governing polymers. RSC Adv.

[26] Yeung CL, Charlesworth S, Iqbal P, Bowen J, Preece JA, Mendes PM (2013). Different formation kinetics and photoisomerization behavior of self-assembled monolayers of thiols and dithiolanes bearing azobenzene moieties. Phys Chem Chem Phys.

[27] Gan Z, Zheng F, Mao W, Zhou C, Chen W, Bach U, Tapping P, Kee TW, Davis JA, Jia B (2019). The optical properties of Cs_4_PbBr_6_–CsPbBr_3_ perovskite composites. Nanoscale.

[28] Sun C, Wei J, Zhao J, Jiang Y, Wang Y, Hu H, Wang X, Zhang Y, Yuan M (2021). Hard and soft Lewis-base behavior for efficient and stable CsPbBr_3_ perovskite light-emitting diodes. Nanophotonics.

[29] Jiang J, Sun X, Chen XC, Wang BW, Chen ZZ, Hu Y, Gio YW, Zhang LF, Ma Y, Gao L, Zhanf FS, Jin L, Chen M, Ma ZW, Zhou YY, Padture NP, Beach K, Terrones H, Shi YF, Gall D, Lu TM, Wertz E, Feng J, Shi J (2019). Carrier lifetime enhancement in halide perovskite via remote epitaxy. Nat Commun.

